# Using machine learning methods to predict electric vehicles penetration in the automotive market

**DOI:** 10.1038/s41598-023-35366-3

**Published:** 2023-05-23

**Authors:** Shahriar Afandizadeh, Diyako Sharifi, Navid Kalantari, Hamid Mirzahossein

**Affiliations:** 1 Department of Transportation, School of Civil Engineering, Iran University of Science and Technology, Tehran, Iran; 2 AECOM, Glen Allen, VA USA; 3 Department of Civil-Transportation Planning, Faculty of Technical and Engineering, Imam Khomeini International University, Qazvin, Iran

**Keywords:** Environmental social sciences, Engineering

## Abstract

Electric vehicles (EVs) have been introduced as an alternative to gasoline and diesel cars to reduce greenhouse gas emissions, optimize fossil fuel use, and protect the environment. Predicting EV sales is momentous for stakeholders, including car manufacturers, policymakers, and fuel suppliers. The data used in the modeling process significantly affects the prediction model’s quality. This research’s primary dataset contains monthly sales and registrations of 357 new vehicles in the United States of America from 2014 to 2020. In addition to this data, several web crawlers were used to gather the required information. Vehicles sale were predicted using long short-term memory (LSTM) and Convolutional LSTM (ConvLSTM) models. To enhance LSTM performance, the hybrid model with a new structure called “Hybrid LSTM with two-dimensional Attention and Residual network” has been proposed. Also, all three models are built as Automated Machine Learning models to improve the modeling process. The proposed hybrid model performs better than the other models based on the same evaluation units, including Mean Absolute Percentage Error, Normalized Root Mean Square Error, R-square, slope, and intercept of fitted linear regressions. The proposed hybrid model has been able to predict the share of EVs with an acceptable Mean Absolute Error of 3.5%.

## Introduction

Emissions of greenhouse gases are increasing rapidly worldwide. According to a United States Environmental Protection Agency report released in 2020, the transportation sector produces about 27% of the entire greenhouse gas emissions in the country, which, compared to other sectors, transportation emits the most greenhouse gases^1^. EVs were introduced as an alternative to gasoline and diesel cars to reduce air pollution and greenhouse gas emissions, optimize the use of natural energy resources and protect the environment. Using electricity generated from renewable energy sources such as wind, water and sunlight for EVs can be one of the most efficient solutions to reduce emissions and climate change^2^. Although much time has passed since the invention of EVs, internal combustion vehicles are still the most popular. EV sales have been on the rise, and in January 2017, the total number of EVs sold worldwide reached two million^3^. Globally, EV sales accounted for 9% of the car market in 2021, a fourfold increase from 2019^4^.

Designing and producing vehicles is time-consuming and requires much investment, so by predicting the number of sales, automobile companies can optimize production, Furthermore, by accurately predicting the penetration of EVs in the market, it is possible to estimate their impact on reducing pollution in the coming years, which is very important from an environmental standpoint. Forecasting the sale of EVs and their penetration into the automotive market has been a significant issue for governments, policymakers, and car manufacturers to plan the production of EVs, set proper policies, and provide sufficient energy and infrastructure.

The main goal of this research is to apply Machine Learning (ML) methods to build an efficient prediction model to estimate the sale of all vehicles in the dataset, the share of EVs in each segment, and determine the main factors that influence the sales of each EV. The effect of a limited number of influencing factors on vehicle sales was examined in previous studies using different models. For this study, a wide range of information was collected, including all factors that previous studies have proven are related to car sales, and it was used in modeling. LSTM and ConvLSTM, powerful Deep Learning (DL) models, have been used for predicting vehicle sales. By combining the two-dimensional Attention model and the Residual network as the proposed hybrid model, it has been tried to improve the performance of the LSTM model. Additionally, using the collected information and the model sensitivity analysis, it was attempted to determine the most influential factors on the sale of each EV.

The literature review of this study includes two general sections. The first section examines ML methods used to predict vehicle sales, and the second section provides an overview of the features used in other methods to predict EV sales.

### ML methods in predicting vehicle sales

Several studies have used ML methods to predict the sales of EVs as time-series data. Multiple Linear Regression and Support Vector Machine (SVM) models were compared for predicting vehicle sales using yearly, quarterly and monthly data (the number of new automobile registrations, the number of automobile sales, and economic indicators such as Gross Domestic Product (GDP), Available Personal Income, Consumer Price Index, Interest Rate, Unemployment Rate, Industrial Investment Demand, Petroleum Charge, Private Consumption, and Latent Replacement Demand) in a study by Brühl et al.^5^ According to the results, the SVM model had better performance based on the error values (Mean Absolute Error and Mean Absolute Percentage Error), was more interpretable, and gave better results based on quarterly data. In the study of Wang et al. ML techniques were used to predict car sales based on sales quantity, economic indicators, wholesale population, unemployment rate, exchange rate, the prices of vehicles, the oil prices, and the prices of vehicle components. Based on evaluation units (R-square and Mean Squared Error), they evaluated the prediction quality of adaptive network-based fuzzy inference system (ANFIS), Artificial Neural Networks (ANN), and autoregressive integrated moving average models; the results showed that ANFIS performed better than the other models^6^. In another study, Hülsmann et al. compared the performance of linear models, such as Ordinary Least Squares and Quantile Regression, against ML methods like SVM, Decision Tree, k–Nearest Neighbor, and Random Forest for predicting vehicle sales. Based on the monthly data of vehicle sales, new car registrations, and economic indicators (such as GDP, Personal Income and Dow Jones), the Decision Tree of ML methods performed better than the other models based on Mean Absolute Percentage Error (MAPE)^7^.

Moreover, Kitabci et al. analysed the impact of economic policies on vehicle sales in Turkey as a macro-environmental factor by multiple regression and neural network methods. They assessed factors such as the vehicle loan rate presented by the banks, the income of the consumers, the tax deductions made by the government for the automobile, the inflation rate, automobile prices, the euro exchange rate, oil prices, and advertisements spent by the businesses. According to the results, neural networks were more accurate in predicting sales than regression models; some factors, including the euro exchange rate, the rates of vehicle loans offered by banks, and the government's tax deductions, have influenced automobile sales^8^. In another research, Bas et al. applied classification ML methods to predict EV adoption using ride-sourcing factors, underlying sociodemographics, and vehicle characteristics; they examined the contributions of different factors to predict outcomes using a method called “Local Interpretable Model-Agnostic Explanations”. Based on the study’s findings, ML models produced highly accurate predictions regarding EV adoption, and the frequent usage of ride-sourcing, knowledge about EVs, and environmental protection awareness were significant factors in explaining the tendency to adopt EVs^9^. In addition, Zhang et al. applied Singular Spectrum Analysis as a univariate time-series model and the Vector Auto-Regression model (VAR) as a multivariate model for forecasting EV sales. According to the results, the VAR model can significantly improve the prediction accuracy because it considers the effect of economic indicators, such as consumer prices, consumer confidence, producers' prices, fuel and vehicle prices, and Baidu data (An indicator of consumer interest and curiosity in EVs)^10^.

In another study, Kaya et al.^11^ used the exchange rate, the GDP, the Consumer Confidence Index, the Consumer Price Index data and a Deep Neural Network model to predict vehicle sales; the results revealed that this ML model predicted sales accurately (based on Mean Squared Error). In another research, Xia et al. introduced the ForeXGBoost model, a vehicle sales prediction system based on large-scale datasets containing comprehensive vehicle information, including brand ID, model, engine power, and displacement. Based on Logarithmic Difference Square Root, MAPE, and running time, the XGBoost model outperforms benchmark algorithms like Linear Regression and Gradient Boosting Decision Trees^12^. Using online survey data and ML methods such as SVM, ANN, Deep Neural Networks, Gradient Boosting Models, and Random Forests, Bas et al. compared different methods for classifying potential EV buyers and identifying the features that affect the adoption of EVs. Results showed that the SVM model outperforms the other algorithms; having only partial information (e.g. only socioeconomic factors) reduces model performance, while synergy across multiple variables increases accuracy^13^. Additionally, Saxena et al. present a study that examines the use of deep learning-based models, including Autoregressive Integration Moving Averages and LSTM models, to predict future directions of vehicle sales. Based on the implementation results, the MAE and the Root Mean Square Error for LSTM-based time series forecasting were reduced, and this model could accurately predict green vehicle sales^14^.

### Factors affecting the sale of EVs in other methods

Developing policies requires understanding users' behavior and prioritizing their choices. Therefore, some previous studies used survey data to predict EV demand. To assess the potential demand for EVs, Beggs et al.^15^ used survey data and vehicle specifications, such as seat capacity, maximum speed, purchase price, and operating costs. In a similar study, the demand for EVs was estimated based on consumer preferences for vehicle attributes by Calfee et al.^16^ The results of this research have shown that the weak performance of EVs limits their demand; however, if EVs become significantly more advanced than other cars or if gasoline becomes scarce, the demand for these vehicles will increase.

Predicting the future demand for EVs is a complex issue. As most studies for new technologies rely on survey data, market share predictions will reflect the share in the survey data, not the actual market share. Consumer opinions and the news published about EVs also influence the sales of these vehicles. Based on Mau et al.^17^ research, EV sales are impacted by published information about the penetration rate of EVs, known as the “The neighbor effect”. Electric vehicles' specifications are another factor affecting their sales. According to Balducci et al.^18^ study to assess plug-in hybrid EV penetration scenarios in the auto market, fuel economy and reduced motor vehicle emissions are the most important factors when purchasing hybrid EVs, while insufficient engine power, high price, and unreliability are the most important reasons for not purchasing these vehicles. Furthermore, Hess et al. used vehicle specifications such as purchase price, vehicle purchase incentives, Miles Per Gallon (MPG) or equivalent, fuel cost per year, fuel availability, refueling time, driving range, maintenance cost per year, and acceleration to explore consumers' preferences in choosing the type of vehicle and the type of fuel. The results have shown that consumers' choices are adversely affected by factors such as purchase price, operating cost, and vehicle age, whereas their choices are positively affected by factors such as better vehicle acceleration, purchase incentives, driving range, and fuel availability^19^.

The sale of EVs is also affected by improving vehicle engine performance and reducing fuel consumption. Using a discrete choice model, Bas et al. investigated EV penetration in the face of new technology for reducing fuel consumption. Results demonstrated a clear tradeoff between the cost of a gasoline-powered system and the fuel savings it provides is perceived by potential purchasers^20^. However, potential EV purchasers are not in this category since their cost–benefit analysis is adverse due to the low cost of electricity^20^. Also, the estimated market shares give a significant share of the market to alternatives that include technology to reduce consumption, due to a more favorable attitude toward environmentally friendly technologies^20^. Additionally, Shafiei et al. analysed the impact of factors such as fuel prices, vehicle attributes, consumer preferences, and social influences on the market share of EVs. The results showed that the combination of high gasoline prices, decreasing EV prices, dropping tax on EVs and eliminating consumer concerns about recharging has the most significant effect on the market share of EVs^21^. Kinski et al.^22^ research shows that the information related to searching on the Internet (Google Trends) for vehicles has a positive and significant relationship with car sales.

Based on the previous research, the following two general conclusions were reached:

- Firstly, ML and DL methods have been proven to be effective at predicting vehicle sales. Therefore, LSTM and ConvLSTM, powerful DL models, have been used for predicting vehicle sales in this research. Furthermore, a hybrid model was also proposed, and all three models were compared in terms of performance.
- Secondly, factors and features that affect EV sales have been identified, and these features have been collected and used in this research.

## Methodology

Artificial Intelligence (AI) refers to the ability of machines to perceive, synthesize, and infer information, as opposed to animals and humans displaying intelligence^23^. Machine learning, artificial neural networks, and deep learning are important tools in the development of AI systems and have been shown to perform well in predicting time series data such as vehicle sales. Recurrent neural networks (RNNs) are a type of neural network that remember what they have already processed and can learn from previous iterations^24^. In other words, an RNN is a class of ANNs where connections between nodes form a directed graph along a temporal sequence; this allows it to exhibit temporal dynamic behavior^24^.

### LSTM

Hochreiter and Schmidhuber introduced the LSTM network, a RNN capable of learning long-term dependencies and predicting sequential data with great accuracy^25^. An LSTM is an extension of an RNN, capable of learning patterns from long sequences of source data by retaining a long-term memory^25^. LSTMs improved the forgetfulness of RNNs. An RNN could retain a memory, but only for its immediate past. An LSTM, on the other hand, introduces loops to generate long-term gradients. While going through its loops, it can discover long-term patterns^25^. LSTM is good at storing past information and performing well when faced with vanishing gradient issues. During ANN training, each weight of the neural network receives an update proportional to the partial derivative of the error function. Vanishing gradients occur when gradients become vanishingly small, effectively preventing the weight from changing^26^.

LSTM can tie together three pieces of information at each time step: the current input data, the short-term memory it receives from the previous cell (the hidden state), and the long-term memory from cells farther away (the cell state)^27^. The LSTM unit consists of an input gate a forget gate, an output gate, and a cell state. The input gate determines how much information should be transferred from the current candidate cell state to the current cell state. The forget gate determines how much historical information should be ignored from the previous cell state. The output flow from cells to the rest of the network can be controlled through the output gate. By regulating the flow of information through the three gates, important information over time intervals can be remembered. According to Eqs. 1–6, the LSTM unit process data in cell state and gates^27^. Reference^27^ provides more details.

1 $$f_{t} = \sigma \left( {W_{fx} x_{t} + W_{fh} h_{t - 1} + b_{f} } \right)$$

2 $$i_{t} = \sigma \left( {W_{ix} x_{t} + W_{ih} h_{t - 1} + b_{i} } \right)$$

3 $$\tilde{C}_{t} = tanh\left( {W_{Cx} x_{t} + W_{Ch} h_{t - 1} + b_{C} } \right)$$

4 $$C_{t} = f_{t} \odot C_{t - 1} + i_{t} \odot \tilde{C}_{t}$$

5 $$o_{t} = \sigma \left( {W_{ox} x_{t} + W_{oh} h_{t - 1} + b_{o} } \right)$$

6 $$h_{t} = o_{t} \odot {\text{tanh}}\left( {C_{t} } \right)$$

**Figure 1:**
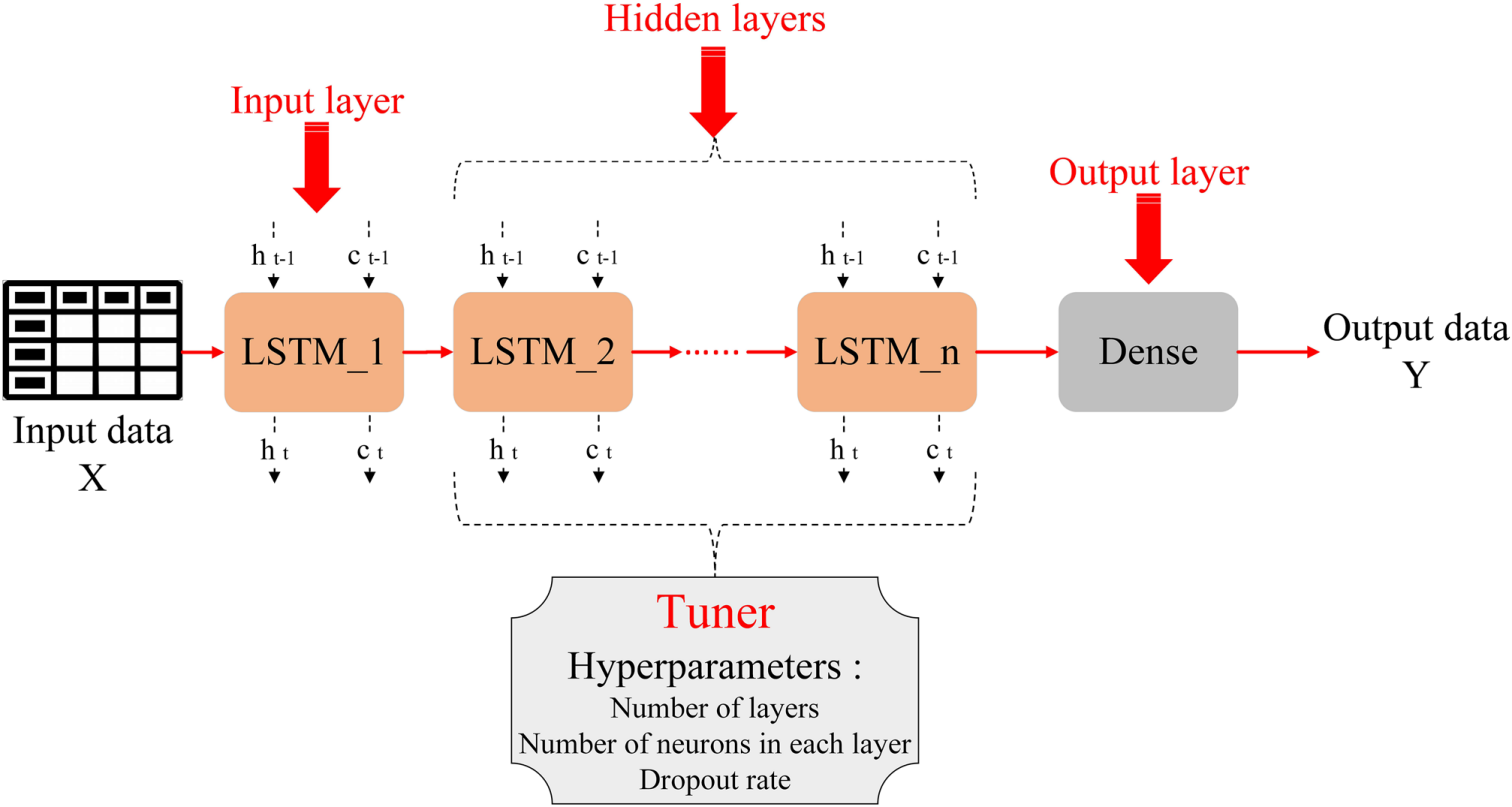
Architecture of the LSTM model.

In the above equations, $f_{t}$, $i_{t}$, and $o_{t}$ are the forget, input, and output gates, respectively; $C_{t}$, $C_{t - 1}$, and $\tilde{C}_{t}$ are the current, previous, and candidate cell state; $\sigma$ and tanh denotes sigmoid and hyperbolic tangent activation functions, respectively; the interconnected weight matrices for each gate and cell state are $W_{fh}$, $W_{ih}$, $W_{oh}$, $W_{Ch}$, respectively; $W_{fx}$, $W_{ix}$, $W_{ox}$, $W_{Cx}$ represent the input weight matrices in the three gates and the cell state, respectively; $b_{f}$, $b_{i}$, $b_{o}$, $b_{C}$ represent the respective bias terms; the Hadamard (element product) product of a matrix is denoted by $\odot$^27^. According to Fig. 1, the input layer is an LSTM layer with the same number of neurons as the input data features. In the next step, one or more LSTM layers are set as the hidden layers, and in the final step, a Dense layer with the ReLU activation function is set as the output layer.

### ConvLSTM

**Figure 2:**
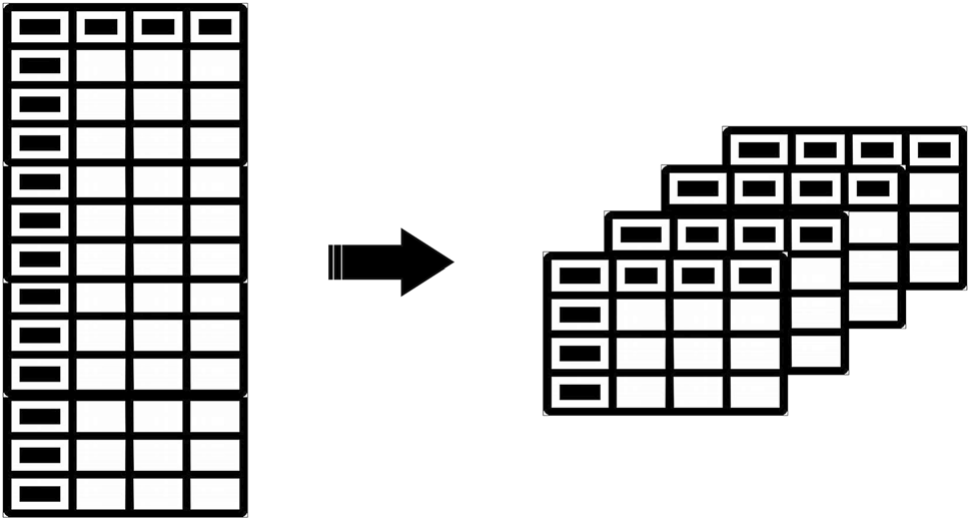
Transforming 2-D matrix into 3-D tensor.

The LSTM model is powerful for handling temporal correlation. In addition, when working with time series data with numerous features, LSTM model performance can be improved by converting the two-dimensional data to a three-dimensional tensor (Fig. 2 illustrates this), connecting states, and applying convolutional operations; this idea was the reason for creating the ConvLSTM model^28^. The ConvLSTM neural network is a fully connected LSTM network with a convolutional structure inside the LSTM cell, which does well in predicting data with temporal correlation. ConvLSTM provides a fully connected extension for data transfer between states and from inputs to states^28^. In other words, ConvLSTM determines the future state of each cell in the grid based on its inputs and neighbours' past states; this can be done by using a convolution operator in the state-to-state and input-to-state transitions^28^. In the ConvLSTM model, data in the input unit, the outputs of each cell, the hidden units, and the gates are arranged as three-dimensional tensors. ConvLSTM has similar parameters as LSTM, and the difference is in how data is transferred and convolutional multiplication is used in calculations, as expressed in Eqs. 7–11^28^. Reference^28^ provides more details.

7 $$f_{t } = \sigma \left( {W_{fx} * x_{t} + W_{fh} * h_{t - 1} + W_{fc} \odot C_{t - 1} + b_{f} } \right)$$

8 $$i_{t } = \sigma \left( {W_{ix} * x_{t} + W_{ih} * h_{t - 1} + W_{ic} \odot C_{t - 1} + b_{i} } \right)$$

9 $$C_{t } = f_{t} \odot C_{t - 1} + i_{t} \odot tanh\left( {W_{cx} * x_{t} + W_{ch} * h_{t - 1} + b_{c} } \right)$$

10 $$o_{t } = \sigma \left( {W_{ox} * x_{t} + W_{oh} * h_{t - 1} + W_{oc} \odot C_{t} + b_{o} } \right)$$

11 $$h_{t } = o_{t} \odot {\text{tanh}}\left( {C_{t} } \right)$$

**Figure 3:**
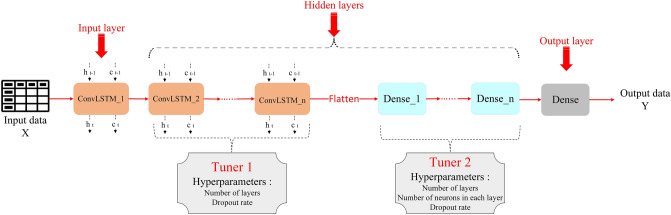
Architecture of the ConvLSTM model.

In ConvLSTM equations, * indicates the convolution operator, and $\odot$ indicates the Hadamard product. As shown in Fig. 3, the input layer is a ConvLSTM layer, the hidden layers are Dense and ConvLSTM layers, and the output layer is a Dense layer with the ReLU activation function.

### Hybrid LSTM with two-dimensional attention and residual network

Time series data have a meaningful temporal relationship. In this research, the data were transformed into three-dimensional tensors with a seven-month time window to maintain the temporal relationship; how to transform a two-dimensional matrix to a three-dimensional tensor is shown in Fig. 2. As an innovation, the “Two-Dimensional Attention” method has been proposed in this research to determine the importance of each car's feature in a seven-month time frame and to use the weighted data in the modeling process. The two-dimensional attention method assigns weights to each feature in the time window based on how much it influences the model, allowing the features with a more significant impact to receive more attention and reduce the model's complexity. The one-dimensional attention model was proposed for the first time by Bahdanau to address the problem of the limited access of the decoder to the model's input information when the encoder vector has a fixed length in the translation machine^29^.

**Figure 4:**
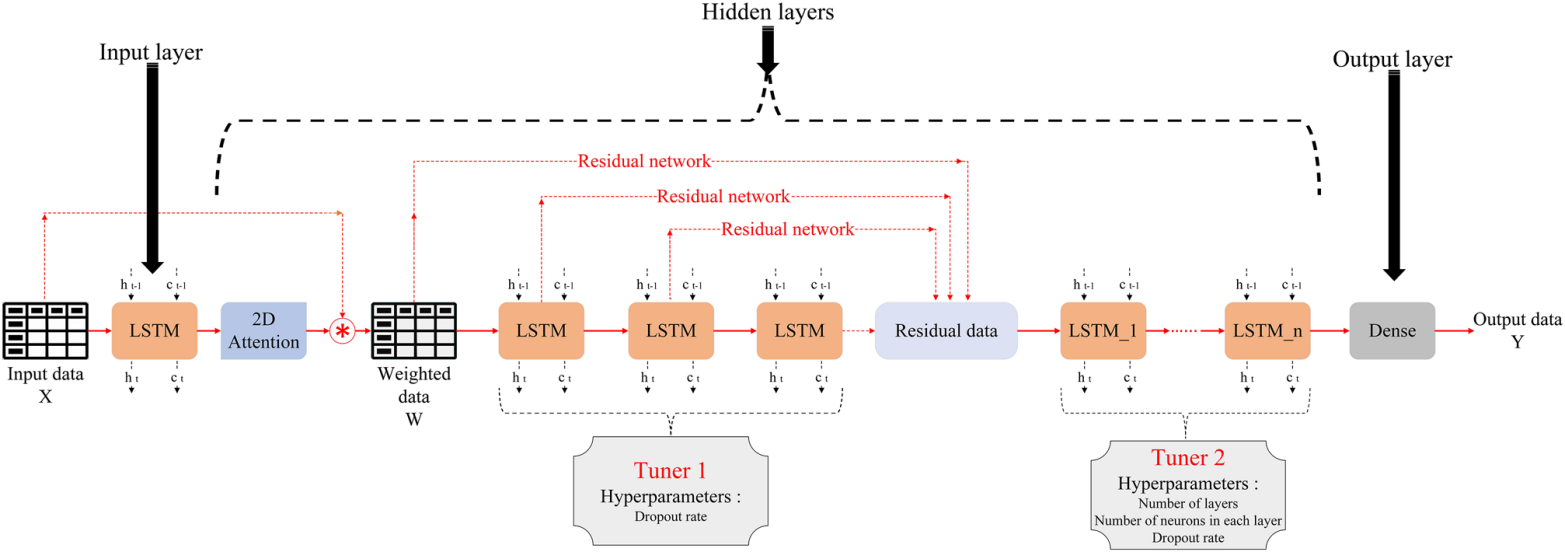
Primary architecture of the hybrid model.

In the LSTM model architecture, which is shown in Fig. 1, several LSTM layers are placed inside the hidden layer. When the number of LSTM layers in the hidden layer increases, the primary layers (the layers adjacent to the input layer) have a lesser effect on the output. The primary layers have processed the input data and learned the relationship between the data well, which is why it has been tried to improve this problem by using the Residual network in the proposed hybrid model. Using the Residual Network, the weighted data and outputs of the primary layers have been transferred to the final layers in the proposed hybrid model, as shown in Fig. 4.

In this study, each input $x$ is represented by an $m \times n$ matrix, where m corresponds to the previous months in the window (7), and n represents the number of vehicle features. After entering the data into the first LSTM layer, the processing is done according to Eqs. 1–6, and the encoded hidden unit ($h$) with the exact dimensions ($m \times n$) is entered into the Attention layer. After that, the alignment score is calculated according to Eq. (12).

12 $$e_{i, j} = {\text{tanh}}\left( {W_{a} \odot h + b_{a} } \right)$$

In Eq. (12), $e_{i, j}$ represents the alignment score, $W_{a}$ is the attention model’s weight (as a trainable variable), $h$ is the encoded hidden unit of the primary LSTM layer, $b_{a}$ is the attention model's bias (as a trainable variable), and the sign "*" denotes the Hadamard product. Since the input data for the attention layer has been encoded by an LSTM layer using tanh nonlinear activation function, tanh has also been used in the attention layer to facilitate data reading during decoding. Each input data element was assigned a degree of attention using Eq. (13).

13 $$\alpha_{i, j} = \frac{{{\text{exp}}\left( {e_{i, j} } \right)}}{{\mathop \sum \nolimits_{i = 1}^{m} \mathop \sum \nolimits_{j = 1}^{n} {\text{exp}}\left( {e_{i, j} } \right)}}$$

Multiplying attention matrix $\alpha_{i, j}$ by raw data matrix $x_{i, j}$ yields a weighted data matrix $W_{i, j}$ based on Eq. (14). The sign “*” denotes the Hadamard product.

14 $$W_{i, j} = \alpha_{i, j} \odot x_{i, j}$$

Weighted data $W_{i, j}$ is then passed through three layers of LSTM as a Residual Network; the output of each layer is combined with the weighted data at the end of the Residual Network and entered into one or more LSTM layers. A Dense layer with the ReLU activation function is the output layer. An overview of the model's architecture is illustrated in Fig. 4.

Other architectures have also been tried in the hybrid model structure, but they were not more efficient, so only the best architecture has been mentioned.

## Data

In this study, EVs are considered as vehicles that use electric motors for propulsion and include all types of EVs. In predicting the sale of vehicles, the number of vehicles in the warehouses is an influential factor, which was not used in this modeling due to a lack of access. Since ML models are based on training, in this study, the models can predict the sales of vehicles that have been on the market for at least 24 months. Emerging vehicles (vehicles that have been on the market for less than 24 months) and cars that have not yet entered the market were not included in the modeling due to insufficient data to train the model. Therefore, the share of EVs in the Automotive Market is expressed as a share in vehicle segments and not as a share of EVs overall.

A wide range of information related to car sales has been used in this research. In the primary dataset, all the data is related to new cars, not used cars. The primary dataset contains monthly information about 357 vehicles, such as brand (or "make" in auto industry lingo, e.g., Benz), model, segmentation, category, shoppers, and sales of different types of cars in the United States from 2014 to 2020. Other information has been extracted based on the cars in this dataset. The data before the outbreak of Covid-19 disease were used since this disease had adverse impacts on the global economy.

As stated in previous studies, vehicle specifications are very effective in car sales prediction models. Vehicle specifications are changed annually. According to Alexa rating^30^ and the comprehensiveness of the information presented on the “Thecarconnection” website^31^, vehicle specifications were collected through this website. In order to save time and automate the collection of information due to a large number of vehicles and changes in specifications of vehicles over time, several web crawler have been designed and used in Python programming language to collect vehicle information. Several vehicle specifications of the "CAR-MID/FULL SIZE" segment are shown in Table 1.

**Table 1:** Annual average values for cars in the “CAR—MID/FULL SIZE” segment.

Segment	Year	Shoppers	Sales	Max price ($)	Min price ($)	Mean price ($)	Max MPG	Min MPG	Mean MPG
CAR—MID/FULL SIZE	2014	50669.8	11031.9	37842.3	23758.7	30238.7	30.9	23.8	26.9
CAR—MID/FULL SIZE	2015	42680	11020.2	39433.6	24456.2	31463.2	31.7	24	27.5
CAR—MID/FULL SIZE	2016	35303.7	10228.3	41084.6	26283.1	33286.7	34.2	25.9	30.1
CAR—MID/FULL SIZE	2017	27345.9	7863.4	41514.6	26594.6	33860.4	41.9	30.2	35.6
CAR—MID/FULL SIZE	2018	26844.8	6841.8	42868.9	26731.4	34492.9	43	30.9	36.6
CAR—MID/FULL SIZE	2019	23699.3	6416.5	43117.1	27358.9	34802.5	43.8	31.7	37.4
CAR—MID/FULL SIZE	2020	22203.4	4704.4	44365.6	27716.9	35750	43.7	32.1	37.4

There is similar information collected for gasoline and EVs; for example, the equivalent MPG in EVs. Price, MPG, max mileage, engine power, and warranty are some of the main features taken into account. Other specifications have been divided into the "safety specifications" and the "other specifications" categories. The safety specifications category includes child safety rear door locks, airbags, ABS brakes, daytime running lights, night vision, driver monitoring alerts, collision mitigation braking system, electronic stability control, and side impact beams. All other features (traction control, fog lamps, tire pressure monitoring, parking sensors, parking assist, and backup cameras) have been transferred to the other specifications category.

The second series of collected data refers to user opinions and news published on reputable websites ranked higher on Alexa^30^. Four websites were examined for this purpose: Autoblog^32^, Auto News^33^, Motor1^34^, and The Car Connection^35^. These websites were crawled using Python web crawlers to save time and collect information automatically. From 2014 to 2020, the daily news published was collected and evaluated for each type of vehicle. The Valence Aware Dictionary and sEntiment Reasoner (VADER) method was used for sentiment analysis of the text. Based on vocabulary analysis, the VADER sentiment analysis method correctly analyzes the sentiment expressed in social media and news texts. Ten independent human raters analyzed over 90,000 ratings in the VADER evaluation, which led to the adoption of 7500 linguistic features that were rated based on their valence scores, which indicate the intensity and polarity of sentiment^36^. For each vehicle, the average monthly score of news and opinions has been calculated based on their daily publication of them.

Another effective source of information about the vehicle market is various economic indicators. Using a Python web crawler, information on several economic indicators affecting the car market has been collected on the Federal Reserve website^37^. Economic indicators include GDP, Consumer Price Index (CPI), Producer Price Index, Consumer Confidence Index, Personal Income Per Capita, Interest Rates on 48-month and 60-month Loans, SP&500, and Dow Jones stock market indicators.

According to Kinski's research, using Google trends in prediction models is beneficial and practical^22^. Three keywords have been selected for Google trend data to evaluate the number of searches for each car from 2014 to 2020 and for the United States of America. The keywords are:

- "Make" + "Model"
- "Price" + "Make" + "Model"
- "Dealer" + "Make"

All cars have the same data collected, and the features collected on a monthly basis for each car are listed in Table 2. Several different trims were available on the market for some vehicles simultaneously, and some characteristics, such as price and MPG, had multiple values for these vehicles. Due to this, the collected values for these characteristics were divided into three categories: minimum, average, and maximum.

**Table 2:** The collected features for each vehicle.

#	Features	#	Features	#	Features
1	Sales*	13	Make and model news score**	25	Mean mileage**
2	Shoppers*	14	Google Trends score 1 (Make and model) **	26	Max engine power**
3	Consumer Confidence Index**	15	Google Trends score 2 (Delaer) **	27	Min engine power**
4	CPI**	16	Google Trends score 3 (Price) **	28	Mean engine power**
5	GDP**	17	Max price**	29	Max safety score**
6	Personal income per capita**	18	Min price**	30	Min safety score**
7	Producer price Index**	19	Mean price**	31	Mean safety score**
8	Dow Jones**	20	Max MPG**	32	Max options score**
9	SP&500**	21	Min MPG**	33	Min options score**
10	Interest Rates on 48-month**	22	Mean MPG**	34	Mean options score**
11	Interest Rates on 60-month**	23	Max mileage**	35	Warranty (mile) **
12	Make news score**	24	Min mileage**		

*Derived from the primary dataset.

**Collected by web crawlers.

The sales feature has been normalized based on the maximum and minimum values from the training data set. Other features are standardized based on each feature's average and standard deviation in the training set. The input data to models are considered seven-month windows to maintain temporal correlation. For example, in the current month, the last seven months' data are input (X), and the current month's sale is output (Y). In order to achieve this, seven-month data matrices were placed consecutively in the third dimension of a three-dimensional tensor.

## Validation and interpretation of results

Since the time series data in this study are monthly, eleven binary columns have been added to the dataset to reflect the effect of each month (in the first month of every year, the column corresponding to the first month is set to 1, and the column for the other months is set to 0). An example of this binary data is shown in Table 3.

**Table 3:** An example of converting month to binary data.

DATE (M/D/Y)	Month 1	Month 2	Month 3	Month 4	Month 5	Month 6	Month 7	Month 8	Month 9	Month 10	Month 11
06/1/2014	0	0	0	0	0	1	0	0	0	0	0
07/1/2014	0	0	0	0	0	0	1	0	0	0	0
08/1/2014	0	0	0	0	0	0	0	1	0	0	0
09/1/2014	0	0	0	0	0	0	0	0	1	0	0
10/1/2014	0	0	0	0	0	0	0	0	0	1	0
11/1/2014	0	0	0	0	0	0	0	0	0	0	1
12/1/2014	0	0	0	0	0	0	0	0	0	0	0

**Figure 5:**
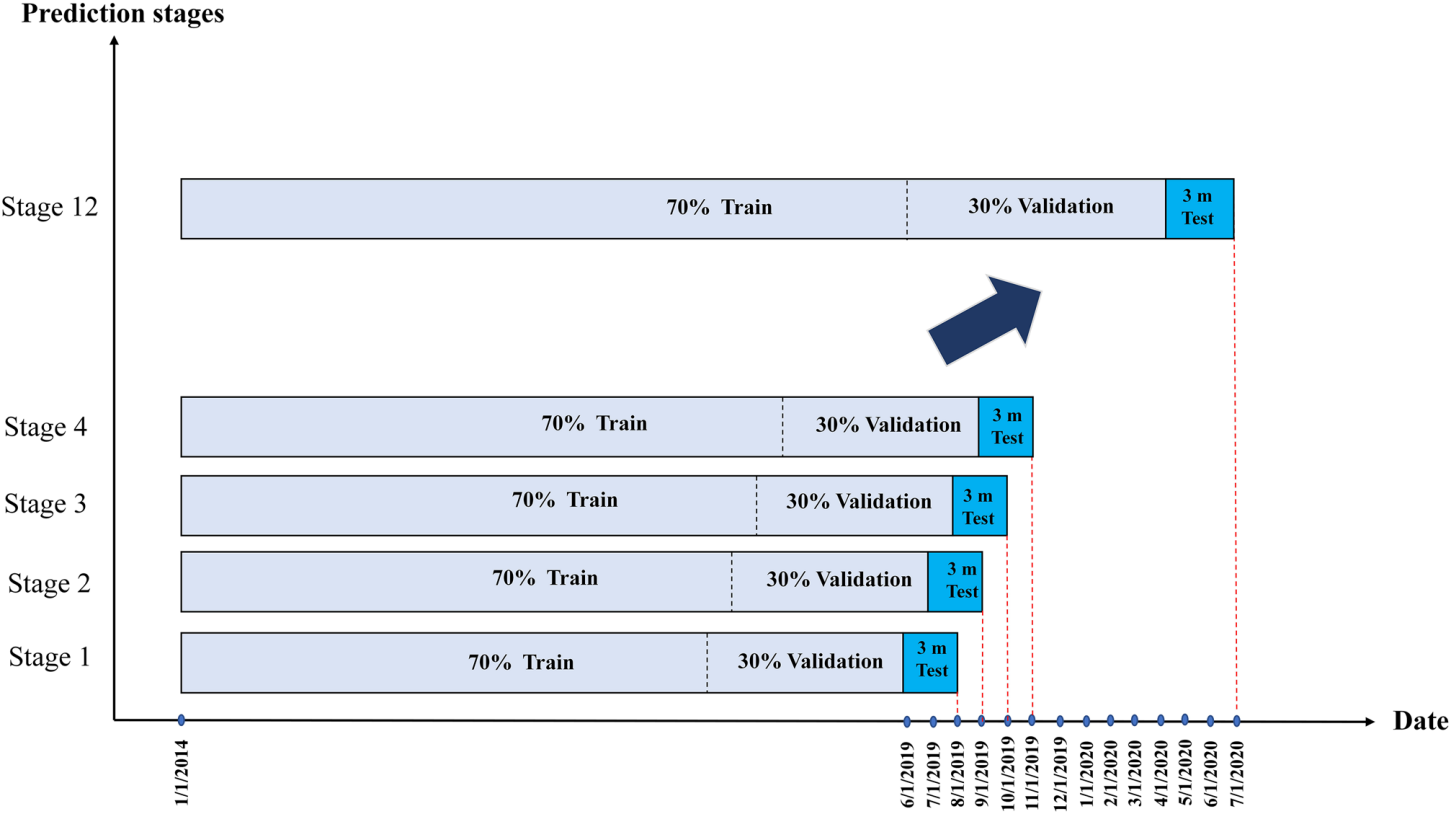
Splitting dataset into training, validation, and testing sets.

For most vehicles, data includes 79 months (January 2014 through July 2020). According to Fig. 5, the last 14 months are selected for the testing set as rolling cross-validation. Using cross-validation on a rolling basis is one way to validate the time-series model. Starting with a subset of data for training, forecasting for later data points and then checking the accuracy of the forecasts. The same forecasted data points are included in the next training dataset, and further forecasts are made.

The model is cross-validated using 12 forecasting stages, with each stage predicting sales in the next three months. During each prediction stage, the preceding months are divided into training and validation (70% for training and 30% for validation. Then these data are transferred to the model, the model predicts sales in the next three months, then the forecast date is moved forward by one month, and this process has been repeated 12 times. Vehicle sales in the next three months are predicted each time the model runs, assuming most of the vehicle's characteristics remain the same. Due to fluctuation and changes in economic conditions, a three-month time horizon is used for predicting the future.

Overfitting is one of the principal problems in ANN training. The Dropout layers between the neural network layers are one of the best solutions in the ANN to avoid overfitting. During the dropout layer, the number of neurons trained in each layer and those discarded is determined randomly (rather than activating all neurons at once, only a fraction are activated)^38^. TensorFlow's early stopping tool is another basic solution to avoid overfitting. Early stopping works in the following way: during the repetition of training, the validation data is used to calculate the error value, and whenever the validation error value increases throughout several epochs, the model is ready to be stopped, and overfitting is prevented. For all three models, both solutions are used to prevent overfitting. Dimensionality reduction is another way to prevent model overfitting. In this study, Principal Component Analysis was used in several modes to reduce dimensions, but this technique was not used due to the significant decrease in model performance.

In order to improve the modeling process, all three models' hyperparameter values and network architectures were determined by Automated Machine Learning (AutoML). AutoML is the process of automating ML applications. The number of hidden layers, the number of neurons in these layers, and the dropout rate was determined by the Tuners. Several values are introduced to the Tuner for each hyperparameter. The Tuner trains different model versions and selects the best one based on the best result (lowest error or loss) on the validation data. This method sets the hyperparameters to the optimal value, and the model is then applied to a test dataset.

The model's error or loss is calculated using the Mean Absolute Error (MAE) loss function in all three models. Selecting a suitable optimization algorithm for the DL model is essential to reduce the run time and reach the desired result. Adam's optimization algorithm is used for these models, which is a generalized version of stochastic gradient descent. It reduces memory usage, converges faster, and corrects high variance and learning rates^39^.

### Comparison of models

With the validation data, hyperparameters are adjusted, and the model is built to predict vehicle sales over the next three months (three months following the last validation date). The model run-time for all vehicles was very long due to the many vehicle types (357). In a random sample of 15 vehicles, different models' states were compared using fixed data, and the results were compared between the three models.

The sale of each vehicle is predicted in 12 stages; each prediction stage includes the prediction for the next three months, respectively, the first month of the prediction, the second month of the prediction, and the third month of the prediction. In total, the first predictions include 12 months, the second predictions include 12 months, and the third predictions include 12 months. Model performance was evaluated using the Mean Absolute Percentage Error (MAPE), the Root Mean Square Error normalized by the change range ($NRSME_{range}$), and the Root Mean Square Error normalized by the mean value ($NRSME_{mean}$) according to Eqs. 15–18.

15 $$MAPE = \frac{100\% }{T} \mathop \sum \limits_{t = 1}^{T} \left| {\frac{{y_{t} - \hat{y}_{t} }}{{y_{t} }}} \right|$$

16 $$RSME = \sqrt {\frac{{\mathop \sum \nolimits_{t = 1}^{T} \left( {y_{t} - \hat{y}_{t} } \right)^{2} }}{T}}$$

17 $$NRSME_{range} = \frac{RSME}{{y_{max} - y_{min} }}$$

18 $$NRSME_{mean} = \frac{RSME}{{y_{mean} }}$$

According to the above equations, $y_{t}$ denotes the actual value at time t, $\hat{y}_{t}$ denotes the predicted value at time t, $y_{max}$ denotes the maximum actual value, $y_{min}$ denotes the minimum actual value, $y_{mean}$ denotes the average actual value, and T is equal to the total number of predicted samples. The average error values of all vehicles were calculated to compare the results of various models. A weighted average was calculated using the total number of sales of each car per month as a weight for the vehicle according to Eq. (19) since the numbers of vehicle sales are not on the same scale, and the error rate is more important in vehicles with high sales. A further method of checking the models' performance is to compare the R-square, slope, and intercept of the linear regressions fitted on predicted and observed data for all three models. Table 4 summarizes the evaluation results of the models.

19 $$\overline{E} = \frac{{\mathop \sum \nolimits_{i = 1}^{N} W_{i} E_{i} }}{{\mathop \sum \nolimits_{i = 1}^{N} W_{i} }}$$

**Table 4:** The comparison of models and their results.

Model	Unite type	Weighted data	Network structure	Prediction months	MAPE	$NRSME_{range}$	$NRSME_{mean}$	Regression 1	Regression 2
LSTM	RNN	No	Direct network	First months	0.256	0.435	0.308	y = 1.149x − 41.627 R^2^ = 0.919	y = 1.146x R^2^ = 0.934
				Second months	0.251	0.456	0.31	y = 1.162x − 41.268 R^2^ = 0.928	y = 1.16x R^2^ = 0.941
				Third months	0.238	0.394	0.283	y = 1.19x − 126.17 R^2^ = 0.956	y = 1.185x R^2^ = 0.963
				Three-month average	0.248	0.428	0.301	–	–
ConvLSTM	RNN-CNN	No	Direct network	First months	0.362	0.583	0.422	y = 0.855x + 968.02 R^2^ = 0.869	y = 0.879x R^2^ = 0.889
				Second months	0.336	0.564	0.387	y = 0.871x + 967.09 R^2^ = 0.895	y = 0.895x R^2^ = 0.910
				Third months	0.344	0.493	0.362	y = 0.923x + 910.72 R^2^ = 0.908	y = 0.947x R^2^ = 0.921
				Three-month average	0.347	0.547	0.39	–	–
Hybrid LSTM with 2-D Attention and Residual network	RNN	Yes	Residual network	First months	0.216	0.336	0.236	y = 1.057x − 41.561 R^2^ = 0.945	y = 1.056x R^2^ = 0.955
				Second months	0.21	0.349	0.235	y = 1.06x − 12.333 R^2^ = 0.947	y = 1.06x R^2^ = 0.956
				Third months	0.183	0.268	0.195	y = 1.059x − 86.411 R^2^ = 0.971	y = 1.057x R^2^ = 0.976
				Three-month average	0.203	0.318	0.222	–	–

In the proposed hybrid model, the error values are lower, the R-square accuracy is higher, the slope value is closer to 1, and the intercept is closer to 0. At this stage, the proposed hybrid model was recognized as preferable to both the LSTM and ConvLSTM models.

### Implementation of the proposed hybrid model to predict the share of EVs

For all vehicles, the proposed hybrid model has been implemented, and 12 points of prediction have been used to determine the sale of all vehicles. Linear regression was fitted on the predicted sales and actual values to evaluate the model's performance, as shown in Table 5.

**Table 5:** Results of fitting the linear regression on the data of all vehicles.

Model	Prediction months	Regression 1	Regression 2
Hybrid LSTM with 2-D Attention and Residual network	First months	y = 0.902x–25.086 R^2^ = 0.912	y = 0.901x R^2^ = 0.934
	Second months	y = 0.888x–26.893 R^2^ = 0.906	y = 0.887x R^2^ = 0.93
	Third months	y = 0.886x–91.764 R^2^ = 0.917	y = 0.881x R^2^ = 0.937

**Figure 6:**
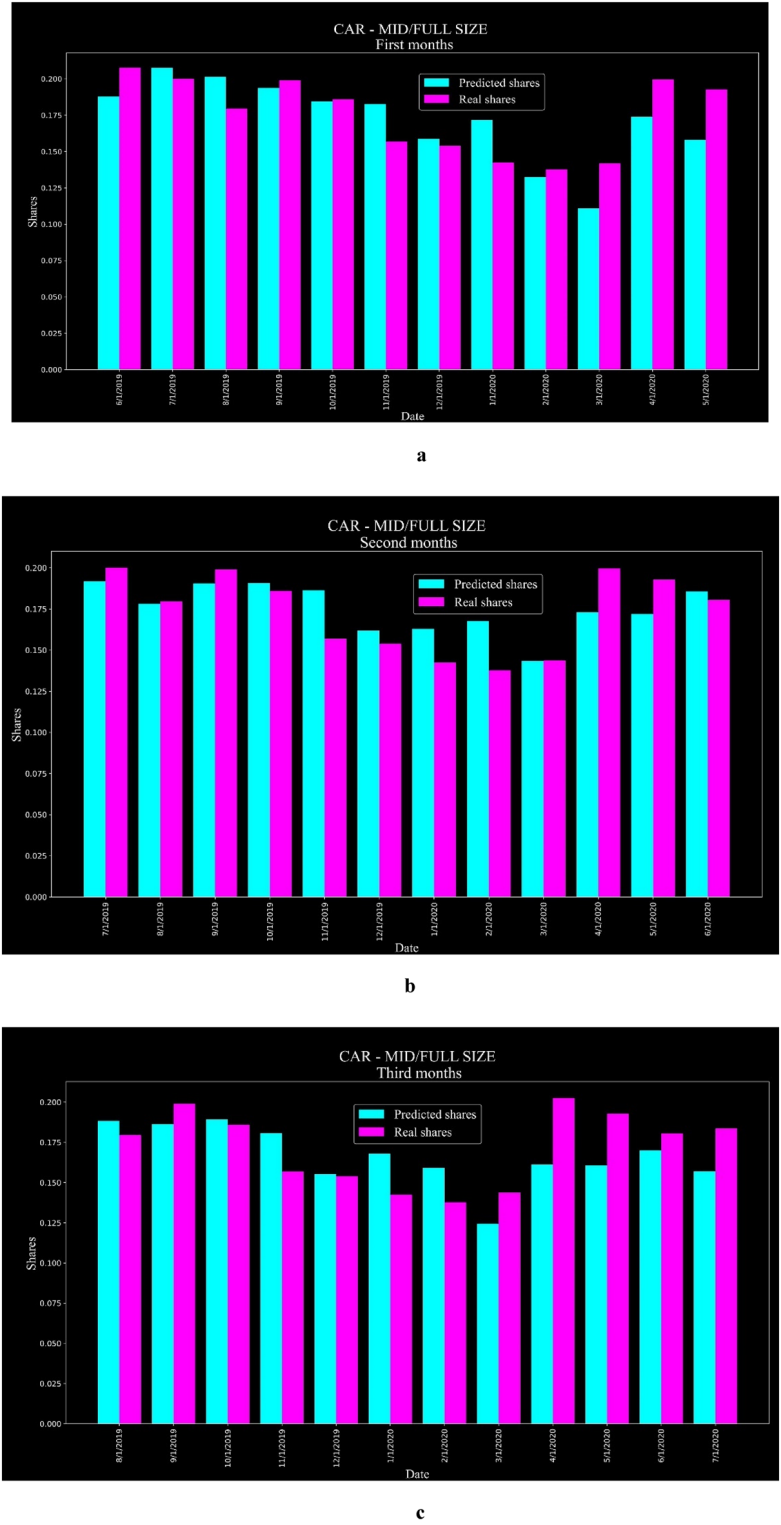
(**a**) Share of EVs in CAR-MID/FULL-SIZE based on the first month of each prediction. (**b**). Share of EVs in CAR-MID/FULL-SIZE based on the second month of each prediction. (**c**) Share of EVs in CAR-MID/FULL-SIZE based on the third month of each prediction.

Primary data segments vehicles by specifications according to segments like CAR-SMALL_COMPACT, CAR-MID_FULL SIZE, MINIVAN LARGE, and PICKUP LARGE. Each segment consists of similar vehicles in appearance and specifications that compete with one another. Segments that include EVs have been separated to determine the share of EVs. Based on actual and predicted sales, the shares of electric and gasoline vehicles have been compared and evaluated for each month of the test data. For example, the CAR-MID/FULL-SIZE segment includes 28 vehicles (23 gasoline vehicles and five EVs). Figure 6 shows the share of EVs in this segment based on twelve prediction stages (three months per stage), separately for the first, second, and third months of each prediction.

All segments' MAEs for EVs' share forecasting in the forecast's first, second, and third months are shown in Table 6. The average MAE value of all segments was calculated as 3.2% for the first months, 3.8% for the second months, and 3.5% for the third months. The average value for all segments and all forecast months was calculated at about 3.5%, which shows that the proposed hybrid model performed well.

**Table 6:** MAE values for all segments that include EVs.

#	Segment	Prediction months	MAE	#	Segment	Prediction months	MAE
1	CAR—LUXURY SMALL/COMPACT	First months	0.086	6	CAR—MID/FULL SIZE	First months	0.018
		Second months	0.09			Second months	0.014
		Third months	0.075			Third months	0.019
2	SUV—LUXURY FULL SIZE	First months	0.022	7	SUV—LUXURY LARGE	First months	0.034
		Second months	0.026			Second months	0.037
		Third months	0.018			Third months	0.037
3	CAR—SUPER LUXURY SPORTS	First months	0.006	8	CAR—SMALL/COMPACT	First months	0.006
		Second months	0.007			Second months	0.008
		Third months	0.006			Third months	0.008
4	CAR—LUXURY MID/FULL SIZE	First months	0.031	9	PICKUP—LARGE	First months	0.016
		Second months	0.044			Second months	0.024
		Third months	0.053			Third months	0.024
5	CAR—LUXURY LARGE	First months	0.057	10	MINIVAN—LARGE	First months	0.046
		Second months	0.063			Second months	0.072
		Third months	0.056			Third months	0.058

As part of the model analysis, the segments that included EVs were separated again and ranked by sales within each segment. The rankings were based on actual sales (actual rank) and predicted sales (predicted rank); the actual rank and predicted rank were used for evaluation. Kendall-Tau correlation (Kendall's correlation) is commonly used to check the concordance of two ranked lists; this technique was used to examine the actual and predicted rankings in this study. Kendall's correlation rate for two rating lists $r_{a}$ and $r_{b}$ ($\tau_{{r_{a} , r_{b} }}$) is represented by Eq. (20) ^40^.

20 $$\tau_{{r_{a} , r_{b} }} = \frac{{n_{c} - n_{d} }}{{\frac{1}{2} n\left( {n - 1} \right)}}$$

In Eq. (20), $n_{c}$ represents the number of concordant pairs, $n_{d}$ represents the number of discordant pairs, and n represents the total number of ranks in each of the rating lists^40^. The maximum number of discordant pairs between two ranking lists equals $\frac{1}{2} n\left( {n - 1} \right)$, and Kendall's correlation equals + 1 if all pairs of ranks are concordant and -1 if none are concordant ^40^. For all segments, Kendall's correlation values were calculated separately for the first, second, and third prediction months, and the average values are shown in Table 7. The average Kendall's correlation value of all segments was calculated as 0.76 for the first months, 0.742 for the second months, and 0.75 for the third months. The average Kendall's correlation value for all segments and all forecast months was calculated at about 0.75, which indicates the great performance of the proposed hybrid model in predicting the ranking.

**Table 7:** Average Kendall's correlation calculated for each segment that includes EVs.

#	Segment	Prediction months	Kendall’s correlation	#	Segment	Prediction months	Kendall's correlation
1	CAR—LUXURY SMALL/COMPACT	First months	0.526	6	CAR—MID/FULL SIZE	First months	0.876
		Second months	0.584			Second months	0.87
		Third months	0.595			Third months	0.87
2	SUV—LUXURY FULL SIZE	First months	0.814	7	SUV—LUXURY LARGE	First months	0.733
		Second months	0.775			Second months	0.7
		Third months	0.791			Third months	0.711
3	CAR—SUPER LUXURY SPORTS	First months	0.917	8	CAR—SMALL/COMPACT	First months	0.716
		Second months	0.95			Second months	0.697
		Third months	0.95			Third months	0.691
4	CAR—LUXURY MID/FULL SIZE	First months	0.789	9	PICKUP—LARGE	First months	0.956
		Second months	0.782			Second months	0.933
		Third months	0.767			Third months	0.956
5	CAR—LUXURY LARGE	First months	0.637	10	MINIVAN—LARGE	First months	0.633
		Second months	0.592			Second months	0.533
		Third months	0.562			Third months	0.517

### Sensitivity analysis

**Figure 7:**
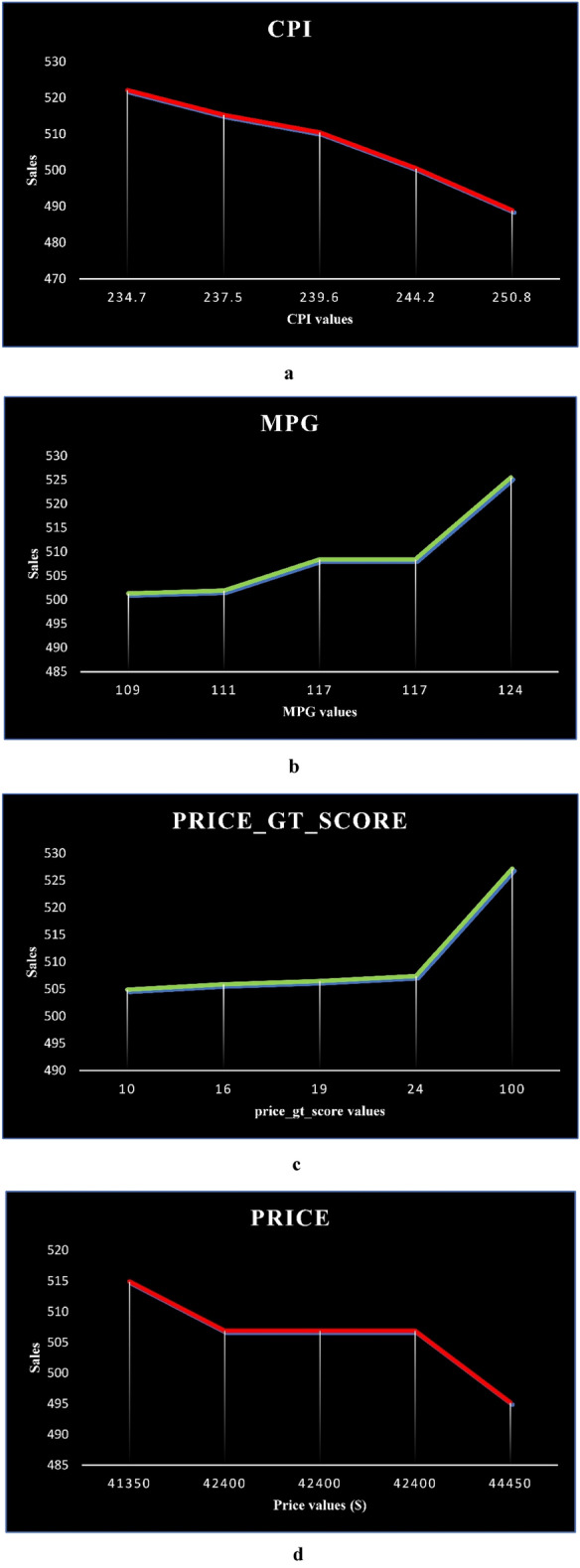
(**a**) Sensitivity analysis plot of influential feature 1 for BMW I3. (**b**) Sensitivity analysis plot of influential feature 2 for BMW I3. (**c**) Sensitivity analysis plot of influential feature 3 for BMW I3. (**d**) Sensitivity analysis plot of influential feature 4 for BMW I3.

Sensitivity analysis was performed to determine which features significantly impacted the trained model. Thus, for each vehicle, the pre-trained model that was evaluated in previous stages has once again predicted the number of vehicle sales with new input data, and its outputs have been assessed. All features, except the investigated feature, are valued at their average. For the investigated feature, the five values from the training data (the min value, the first quartile, the second quartile, the third quartile, and the max value) are taken into consideration. Five predictions were made based on these five values, and a range of changes in predicted sales was calculated. The change ranges for all features have been measured, and the four features with the most extensive range have been identified. As an example, during the sensitivity analysis of the BMW I3 for 2020, the following four features had the broadest range of changes: the Consumer Price Index (CPI), the equivalent MPG for EVs, the Google search score for car prices (Google Trends), and the car price. This EV's sensitivity analysis plots are shown in Fig. 7.

Based on Eq. (21), slope values for the four characteristics with the most extensive range of changes are calculated in different parts of the graph, and the results are summarized in Table 8. For example, the number of sales of this EV has decreased by 8 for every thousand-dollar increase in price when the price is in the range of the minimum value to the first quarter. As the slope is zero percent in the second and third parts of the graph, the price in the first, second, and third quartiles is equal, and when the price is in the third quartile to the maximum price, the number of sales for this EV decrease by 6 for every thousand-dollar increase in price.

21 $$S = \frac{{y_{2} - y_{1} }}{{x_{2} - x_{1} }}$$

**Table 8:** Slope values in sensitivity analysis plots.

Features	S in part 1	S in part 2	S in part 3	S in part 4
CPI	− 2.487	− 2.339	− 2.122	− 1.77
MPG	0.326	1.085	0	2.433
Google Trends score 3 (Price)	0.17	0.182	0.193	0.26
price	− 0.008	0	0	− 0.006

There has been a decrease in car sales due to the increase in the CPI. It is also true that with the increase in the CPI, the final price of the car and the price of auto parts have increased, which has led to a decrease in the desire to buy this car. The second feature is equivalent MPG for EVs, a higher equivalent MPG indicating better performance and less fuel consumption in a fixed distance has led to an increase in sales of this car. The third feature identified is the increase in the car price search score on Google (Google Trend), an indicator that buyers are more curious about this car, contributing to its sales. The fourth specified feature of the car is its price, and its sales have decreased with the increase in its price. As a result of the sensitivity analysis, the manufacturers of this car could use policies such as lowering the price of the car and its parts (CPI and car price), improving the performance of the vehicle's engine (the equivalent MPG), and developing advertisements and introducing the car to the public (Google trend score) to increase sales.

Sensitivity analysis has been conducted for each EV, and the results show different sensitivity for each vehicle. From each segment that includes EVs, one vehicle was selected as a sample, and the results of its sensitivity analysis are shown in Table 9.

**Table 9:** The results of sensitivity analysis for sample EVs.

Make and model	Segment	Feature #1	Feature #2	Feature #3	Feature #4
BMW I3	CAR—LUXURY SMALL/COMPACT	CPI	Max MPG	Google Trends score 3 (Price)	Min price
TESLA MODEL X	SUV—LUXURY FULL SIZE	Make news score	Shoppers	Min price	month-indicating feature (Month_8)
ACURA_NSX_	CAR—SUPER LUXURY SPORTS	Mean safety score	Mean options score	Min price	month-indicating feature (Month_11)
TESLA MODEL 3	CAR—LUXURY MID/FULL SIZE	Sales	Make news score	Max MPG	SP&500
TESLA MODEL S	CAR—LUXURY LARGE	Make news score	Min price	Google Trends score 3 (Price)	CPI
TOYOTA MIRAI	CAR—MID/FULL SIZE	Min price	month-indicating feature (Month_6)	Personal income per capita	CPI
MERCEDES BENZ GLS	SUV—LUXURY LARGE	CPI	Interest Rates on 60-month	Min price	Mean options score
NISSAN LEAF	CAR—SMALL/COMPACT	Shoppers	Personal income per capita	CPI	Min price
Ram 1500	PICKUP—LARGE	Mean options score	month-indicating feature (Month_10)	Sales	Make news score
CHRYSLER PACIFICA	MINIVAN—LARGE	Min price	month-indicating feature (Month_1)	Consumer Confidence Index	Max MPG

Each EV's sensitivity analysis identifies features that differ from the others, as shown in Table 9. According to the results of the sensitivity analysis, ten features that were most frequently found in the sensitivity analysis of all the EVs were identified as the most influential features: Shoppers, Min price, CPI, Sales, Google Trends score 3 (Price), Make & model news score, Personal income per capita, Make news score, Interest Rates on 60-month, and Mean options score, respectively.

## Conclusion

This study addresses an important topic from a business perspective. Car manufacturers can benefit from this research by understanding their market share and the effect of pricing and vehicle specification on the market share. They can use the results of this study to analyze both their EV market as well as their Non-EV market. Lower down the funnel, car dealers that operate in a highly competitive environment can strategize their sales events, marketing campaigns, and discounts to meet their business goals and target sales. Finally, the model enables the public sector to understand the effect of tax policies on the share of EV vehicles in case they like to promote them.

This study used ML methods to develop a prediction model that estimated the sale of all cars in the dataset, the share of EVs in each segment and identified the main factors affecting each EV's sales. In this research, several web crawlers have been used to collect various data, including factors that previous studies have proven to be associated with EV sales. Vehicles sale were predicted using LSTM, ConvLSTM, and the proposed hybrid model (Hybrid LSTM with two-dimensional Attention and Residual network). Several ML tools have been used to improve the model's training and the modeling process, such as transforming two-dimensional time series data into three-dimensional tensors, Dropout layers, early stopping tools, and AutoML. Because of the variety of car types and the long running time of the models, a random selection of fifteen types of cars was made. All three models are evaluated based on the same evaluation units: the MAPE, NRSME_range, and NRSME_mean, R-square, slope, and intercept of fitted linear regressions have also been assessed. The average error values in the three months of prediction were as follows:

- The MAPE value of the proposed hybrid model was 4.5% less than the LSTM model and 14.4% less than the ConvLSTM model.
- The NRSME_range value of the hybrid model was 0.11 less than the LSTM model and 0.22 less than the ConvLSTM model.
- The NRSME_mean value of the hybrid model was 0.079 less than the LSTM model and 0.169 less than the ConvLSTM model.

As a result of fitting linear regressions to the predicted and actual values, for all three months of predictions, the proposed hybrid model has a higher R-square value, its slope is closer to one, and its intercept is closer to zero, which indicates that the hybrid model performed better than the other two. In comparing the models, it was found that the proposed hybrid model conducted better than other models and was selected to predict the sale of all vehicles in the dataset. Based on the linear regression fitted to the predicted sales and the actual sales of all vehicles, the R-square values for the first, second and third prediction months were 0.912, 0.906, and 0.917.

The predicted sales of all vehicles were used to calculate the predicted share of EVs in each segment and compare them with the actual values. Across all segments and forecasting months, the average MAE value for EV share is about 3.5%, and the hybrid model has accurately predicted the share of EVs across all segments. To further analyze the model results, the cars were ranked according to the number of actual and predicted sales within each segment. The average Kendall's correlation value for all segments and all forecast months was calculated at about 0.75, which indicates the high performance of the proposed hybrid model in predicting the ranking.

The sensitivity analysis was performed to evaluate the model further and identify its most influential features. The results have shown that each EV's sensitivity analysis identifies features that differ from the others. According to the sensitivity analysis of the BMW I3 for 2020, the following four features were most affected: the Consumer Price Index, the equivalent MPG for EVs, the Google search score, and the car price. As a result of the sensitivity analysis, the manufacturers of this car could use policies such as lowering the price of the car and its parts, improving the engine's performance, developing advertisements, and better introducing the car to increase sales (See Appendix Tables A1 to A4.2, Fig. A1).

This research has achieved the following accomplishments:

- A wide variety of factors have been collected and used as variables to model the sale of EVs.
- LSTM and ConvLSTM, powerful DL models, have been used for predicting vehicle sales. By combining the two-dimensional Attention model and the Residual network, the performance of the LSTM model was enhanced, and the innovative hybrid model performed better than the other two.
- EVs differ in terms of the most influential factors for sales depending on the sensitivity analysis results. The ten features that appeared the most in the sensitivity analysis of all EVs were identified as the most influential, including Shoppers, Min price, CPI, Sales, Google Trends score 3 (Price), News score for make and model, Personal income per capita, News score for make, Interest Rates on 60-month, and Mean options score, respectively.

## Supplementary Information

Supplementary Information.

## Data Availability

The primary dataset was taken from Autometrics, and other data were collected using web crawlers. The data is available from the corresponding author on reasonable request.
