# Supplementary material for: Using machine learning methods to predict electric vehicles penetration in the automotive market
Source: Sci Rep. 2023 May 23;13:8345. doi: 10.1038/s41598-023-35366-3 (PMC10204681; doi:10.1038/s41598-023-35366-3)
Supplement: Supplementary file 1 — Supplementary Information. [file 41598_2023_35366_MOESM1_ESM.docx]

**Appendices**

**Table A.1.** Acura ILX data in 2014 taken from the primary dataset

| **Make** | **Model** | **Date** | **Segment** | **Category** | **Shoppers** | **Sales** |
| --- | --- | --- | --- | --- | --- | --- |
| ACURA | ILX | 1/1/2014 | CAR - LUXURY SMALL/COMPACT | Car | 17,631 | 1,301 |
| ACURA | ILX | 2/1/2014 | CAR - LUXURY SMALL/COMPACT | Car | 19,737 | 1,683 |
| ACURA | ILX | 3/1/2014 | CAR - LUXURY SMALL/COMPACT | Car | 19,144 | 1,659 |
| ACURA | ILX | 4/1/2014 | CAR - LUXURY SMALL/COMPACT | Car | 20,191 | 1,629 |
| ACURA | ILX | 5/1/2014 | CAR - LUXURY SMALL/COMPACT | Car | 20,364 | 928 |
| ACURA | ILX | 6/1/2014 | CAR - LUXURY SMALL/COMPACT | Car | 23,488 | 1,235 |
| ACURA | ILX | 7/1/2014 | CAR - LUXURY SMALL/COMPACT | Car | 24,733 | 1,798 |
| ACURA | ILX | 8/1/2014 | CAR - LUXURY SMALL/COMPACT | Car | 24,809 | 1,464 |
| ACURA | ILX | 9/1/2014 | CAR - LUXURY SMALL/COMPACT | Car | 27,729 | 1,618 |
| ACURA | ILX | 10/1/2014 | CAR - LUXURY SMALL/COMPACT | Car | 21,823 | 1,652 |
| ACURA | ILX | 11/1/2014 | CAR - LUXURY SMALL/COMPACT | Car | 20,651 | 1,730 |
| ACURA | ILX | 12/1/2014 | CAR - LUXURY SMALL/COMPACT | Car | 25,487 | 992 |

**Table A.2.** Acura ILX Economic Indicators data, in 2014, collected by web crawlers

| **Make** | **Model** | **Date** | **CCI** | **CPI** | **Dow Jones** | **Interest Rates on 48-month** | **Interest Rates on 60-month** | **GDP** | **Personal income per capita** | **PPI** | **SP$500** |
| --- | --- | --- | --- | --- | --- | --- | --- | --- | --- | --- | --- |
| ACURA | ILX | 1/1/2014 | 99.294 | 235.547 | 16278.801 | 4.23 | 4.37 | 17432.909 | 46784 | 123.2 | 1824.581 |
| ACURA | ILX | 2/1/2014 | 99.331 | 236.028 | 15959.502 | 4.5 | 4.46 | 17432.909 | 46784 | 124.8 | 1816.831 |
| ACURA | ILX | 3/1/2014 | 99.414 | 236.468 | 16303.646 | 4.5 | 4.46 | 17432.909 | 46784 | 125.5 | 1862.565 |
| ACURA | ILX | 4/1/2014 | 99.399 | 236.918 | 16373.901 | 4.5 | 4.46 | 17721.657 | 47410 | 125.5 | 1861.081 |
| ACURA | ILX | 5/1/2014 | 99.3685 | 237.231 | 16568.497 | 4.16 | 4.11 | 17721.657 | 47410 | 126.3 | 1889.846 |
| ACURA | ILX | 6/1/2014 | 99.359 | 237.498 | 16847.797 | 4.16 | 4.11 | 17721.657 | 47410 | 126.3 | 1947.709 |
| ACURA | ILX | 7/1/2014 | 99.468 | 237.46 | 16998.571 | 4.16 | 4.11 | 17849.912 | 47931 | 126.7 | 1974.606 |
| ACURA | ILX | 8/1/2014 | 99.702 | 237.477 | 16770.903 | 4.06 | 4.07 | 17849.912 | 47931 | 124.8 | 1961.268 |
| ACURA | ILX | 9/1/2014 | 100.031 | 237.43 | 17106.488 | 4.06 | 4.07 | 17849.912 | 47931 | 124.8 | 1994.471 |
| ACURA | ILX | 10/1/2014 | 100.435 | 236.983 | 16697.492 | 4.06 | 4.07 | 18003.399 | 48512 | 124.6 | 1935.744 |
| ACURA | ILX | 11/1/2014 | 100.882 | 236.252 | 17654.443 | 4.53 | 4.27 | 18003.399 | 48512 | 124.9 | 2045.38 |
| ACURA | ILX | 12/1/2014 | 101.184 | 234.747 | 17768.379 | 4.53 | 4.27 | 18003.399 | 48512 | 123.9 | 2056.451 |

**Table A.3.** News score data and Google Trend score for Acura ILX, in 2014, collected with web crawlers

| **Make** | **Model** | **Date** | **Make news score** | **Make & model news score** | **Google Trends score 1 (Make & model)** | **Google Trends score 2 (Delaer)** | **Google Trends score 3 (Price)** |
| --- | --- | --- | --- | --- | --- | --- | --- |
| ACURA | ILX | 1/1/2014 | 0.139 | 0 | 2 | 63 | 2 |
| ACURA | ILX | 2/1/2014 | 0.092 | 0.034 | 3 | 40 | 1 |
| ACURA | ILX | 3/1/2014 | 0.225 | 0.318 | 2 | 58 | 2 |
| ACURA | ILX | 4/1/2014 | 0.046 | 0.419 | 3 | 48 | 1 |
| ACURA | ILX | 5/1/2014 | 0.206 | 0.212 | 3 | 50 | 2 |
| ACURA | ILX | 6/1/2014 | 0.299 | -0.423 | 3 | 50 | 0 |
| ACURA | ILX | 7/1/2014 | 0.001 | -0.542 | 2 | 49 | 3 |
| ACURA | ILX | 8/1/2014 | 0.056 | 0.192 | 2 | 50 | 3 |
| ACURA | ILX | 9/1/2014 | 0.204 | 0.412 | 3 | 37 | 1 |
| ACURA | ILX | 10/1/2014 | 0.138 | 0.012 | 3 | 49 | 0 |
| ACURA | ILX | 11/1/2014 | -0.097 | -0.197 | 4 | 41 | 2 |
| ACURA | ILX | 12/1/2014 | 0.024 | -0.185 | 3 | 44 | 1 |

**Table A.4.1.** Acura ILX specifications data, in 2014, collected with web crawlers

| **Make** | **Model** | **Date** | **Max price** | **Min price** | **Mean price** | **Max MPG** | **Min MPG** | **Mean MPG** | **Max mileage** | **Min mileage** | **Mean mileage** |
| --- | --- | --- | --- | --- | --- | --- | --- | --- | --- | --- | --- |
| ACURA | ILX | 1/1/2014 | 34,600 | 26,900 | 30,067 | 38 | 25 | 31 | 502 | 330 | 407 |
| ACURA | ILX | 2/1/2014 | 34,600 | 26,900 | 30,067 | 38 | 25 | 31 | 502 | 330 | 407 |
| ACURA | ILX | 3/1/2014 | 34,600 | 26,900 | 30,067 | 38 | 25 | 31 | 502 | 330 | 407 |
| ACURA | ILX | 4/1/2014 | 34,600 | 26,900 | 30,067 | 38 | 25 | 31 | 502 | 330 | 407 |
| ACURA | ILX | 5/1/2014 | 34,600 | 26,900 | 30,067 | 38 | 25 | 31 | 502 | 330 | 407 |
| ACURA | ILX | 6/1/2014 | 34,600 | 26,900 | 30,067 | 38 | 25 | 31 | 502 | 330 | 407 |
| ACURA | ILX | 7/1/2014 | 34,600 | 26,900 | 30,067 | 38 | 25 | 31 | 502 | 330 | 407 |
| ACURA | ILX | 8/1/2014 | 34,600 | 26,900 | 30,067 | 38 | 25 | 31 | 502 | 330 | 407 |
| ACURA | ILX | 9/1/2014 | 34,600 | 26,900 | 30,067 | 38 | 25 | 31 | 502 | 330 | 407 |
| ACURA | ILX | 10/1/2014 | 34,600 | 26,900 | 30,067 | 38 | 25 | 31 | 502 | 330 | 407 |
| ACURA | ILX | 11/1/2014 | 34,600 | 26,900 | 30,067 | 38 | 25 | 31 | 502 | 330 | 407 |
| ACURA | ILX | 12/1/2014 | 34,600 | 26,900 | 30,067 | 38 | 25 | 31 | 502 | 330 | 407 |

**Table A.4.2.** Acura ILX specifications data, in 2014, collected with web crawlers

| **Make** | **Model** | **Date** | **Max engine power** | **Min engine power** | **mean engine power** | **Max safety score** | **Min safety score** | **Mean safety score** | **Max options score** | **Min options score** | **Mean options score** | **Warranty (mile)** |
| --- | --- | --- | --- | --- | --- | --- | --- | --- | --- | --- | --- | --- |
| ACURA | ILX | 1/1/2014 | 201 | 111 | 146 | 0.722 | 0.667 | 0.713 | 0.44 | 0.44 | 0.44 | 50,000 |
| ACURA | ILX | 2/1/2014 | 201 | 111 | 146 | 0.722 | 0.667 | 0.713 | 0.44 | 0.44 | 0.44 | 50,000 |
| ACURA | ILX | 3/1/2014 | 201 | 111 | 146 | 0.722 | 0.667 | 0.713 | 0.44 | 0.44 | 0.44 | 50,000 |
| ACURA | ILX | 4/1/2014 | 201 | 111 | 146 | 0.722 | 0.667 | 0.713 | 0.44 | 0.44 | 0.44 | 50,000 |
| ACURA | ILX | 5/1/2014 | 201 | 111 | 146 | 0.722 | 0.667 | 0.713 | 0.44 | 0.44 | 0.44 | 50,000 |
| ACURA | ILX | 6/1/2014 | 201 | 111 | 146 | 0.722 | 0.667 | 0.713 | 0.44 | 0.44 | 0.44 | 50,000 |
| ACURA | ILX | 7/1/2014 | 201 | 111 | 146 | 0.722 | 0.667 | 0.713 | 0.44 | 0.44 | 0.44 | 50,000 |
| ACURA | ILX | 8/1/2014 | 201 | 111 | 146 | 0.722 | 0.667 | 0.713 | 0.44 | 0.44 | 0.44 | 50,000 |
| ACURA | ILX | 9/1/2014 | 201 | 111 | 146 | 0.722 | 0.667 | 0.713 | 0.44 | 0.44 | 0.44 | 50,000 |
| ACURA | ILX | 10/1/2014 | 201 | 111 | 146 | 0.722 | 0.667 | 0.713 | 0.44 | 0.44 | 0.44 | 50,000 |
| ACURA | ILX | 11/1/2014 | 201 | 111 | 146 | 0.722 | 0.667 | 0.713 | 0.44 | 0.44 | 0.44 | 50,000 |
| ACURA | ILX | 12/1/2014 | 201 | 111 | 146 | 0.722 | 0.667 | 0.713 | 0.44 | 0.44 | 0.44 | 50,000 |

**Figure A.1.** Monthly sales averages for five main categories in the dataset

**
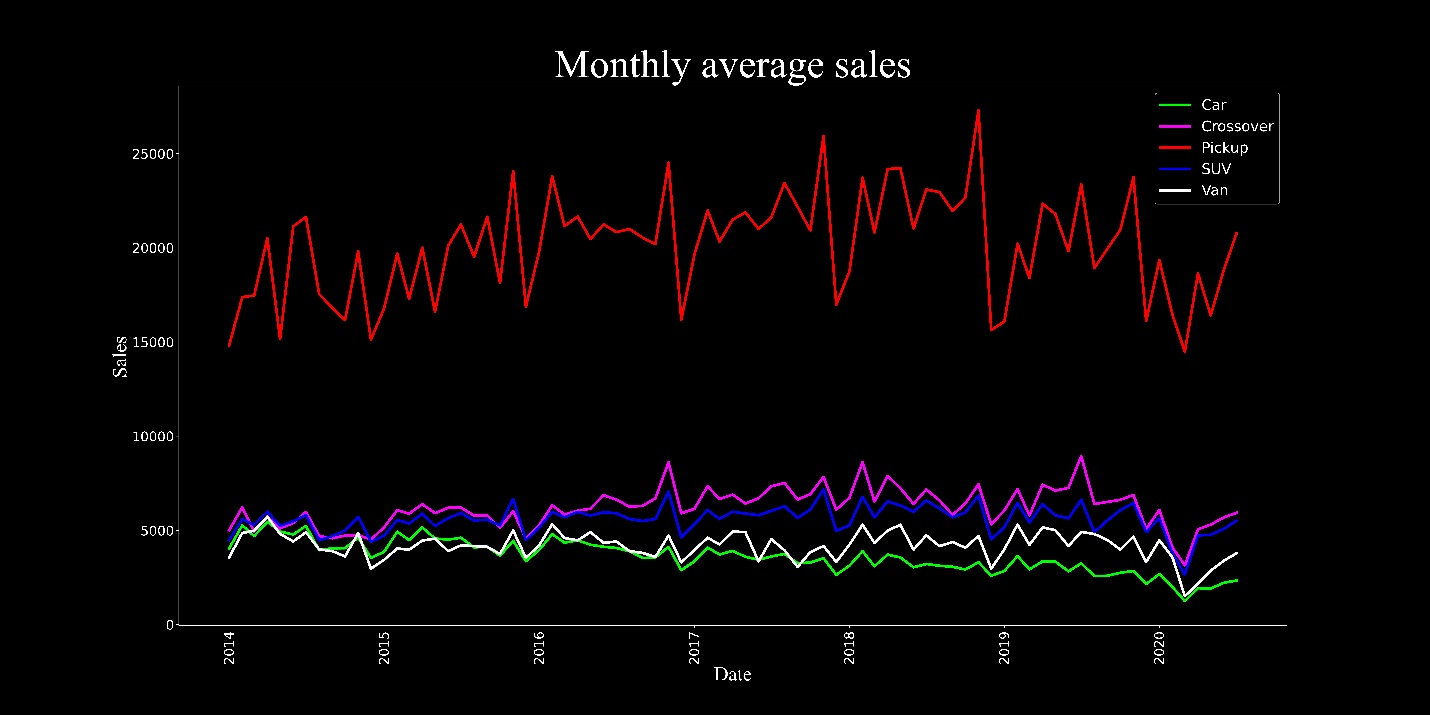
**
